# Editorial: Endocrine and paracrine regulation of spermatogenesis

**DOI:** 10.3389/fendo.2022.984409

**Published:** 2022-07-26

**Authors:** Erwin Goldberg, Polina V. Lishko, Vassilios Papadopoulos, Barry Zirkin

**Affiliations:** ^1^ Department of Molecular Biosciences, Northwestern University, Evanston, IL, United States; ^2^ Department of Molecular and Cell Biology, University of California, Berkeley, Berkeley, CA, United States; ^3^ Department of Pharmacology and Pharmaceutical Sciences, School of Pharmacy, University of Southern California, Los Angeles, CA, United States; ^4^ Department of Biochemistry and Molecular Biology, Johns Hopkins Bloomberg School of Public Health, Baltimore, MD, United States

**Keywords:** endocrine and paracrine regulation of spermatogenesis, endocrine regulation, paracrine regulation, spermatogenesis, testes

## Introduction

Continuous generation of male gametes occurs through the tightly controlled multistep process of spermatogenesis. This process includes the formation and differentiation of spermatogonia, their entry into meiosis, recombination of the paternal genome during meiosis, and the differentiation of the spermatids that result from meiosis into advanced spermatids/spermatozoa. Somatic cell-cell and somatic-germ cell interactions within the testis define the local milieu and endocrine environment driving germ cell development and spermatogenesis. The spermatozoa formed in the testis gain the ability for forward motility and fertility during their passage though the epididymis. The process of spermatogenesis ensures the propagation of the species while also providing biological diversity and adaptation.

This volume, edited by Erwin Goldberg (Northwestern University), Polina Lishko (University of California, Berkeley), Vassilios Papadopoulos (University of Southern California), and Barry Zirkin (Johns Hopkins University), is dedicated to how the process of spermatogenesis is regulated by endocrine and paracrine mechanisms. It encompasses peer-reviewed perspectives, reviews, opinion papers, and original research reports by scientists at the cutting edges of their fields. The authors were invited by the guest editors to submit articles in the broad area of endocrine and paracrine regulation of spermatogenesis. The topics covered in the volume include the regulation of spermatogonial development from stem cells, spermatogonial division to form cells that enter meiosis, the process of meiosis, spermatogenesis and its regulation, the effects of aging and of chemical exposure on sperm formation and function, new approaches to male contraception, and the application of new molecular technologies. The editors have organized the papers into four major areas: Spermatogonia: Development and Regulation; Sperm Formation: Molecular and Hormonal Regulation; Effects of Age and Exogenous Influences on Sperm Formation and Function; and Clinical Applications: Testosterone, Spermatogenesis and Contraception. The following descriptions summarize the contents of the papers contained in each of these four major areas:

## Spermatogonia: Development and regulation

Analyses of altered activin A in murine models by Moody et al. implicate activin A as a key determinant of early germline formation and highlight the potential for altered activin A levels *in utero* to increase the risk of testicular pathologies that arise from impaired germline maturation. Manku et al., building on their previously identified Ubiquitin-Proteasome System (UPS) enzymes that are dynamically altered during gonocyte differentiation, focus on the role of the RING finger protein 149 (RNF149), an E3 ligase expressed in gonocytes and downregulated in spermatogonia. The new data that are presented support a role for RNF149 in gonocyte proliferation.

Wright reviews the *in vivo* regulation of spermatogonial stem cells (SSCs) in adult testes by Sertoli cell-produced glial cell line-derived neurotrophic factor (GDNF) through the use of a novel chemical-genetic approach to diminish replication and increase the differentiation of SSCs. The data that are reviewed suggest that GDNF may prove to be an effective therapy for men whose testes contain only Sertoli cells (SCO syndrome).

Diao et al. summarize research progress on the regulation of spermatogonial stem cells (SSCs), and the potential application of SSCs for fertility restoration. The authors suggest that *in vitro* spermatogenesis from SSCs produced from induced pluripotent stem cells (iPSCs) might be of use in improvement of spermatogenesis.

## Sperm formation: Molecular and hormonal regulation

Leydig cells are the main site of production of the male sex hormone testosterone, a steroid that is critical for the development of male sexual characteristics and spermatogenesis. The commitment, differentiation, and function of Leydig cells require the coordinated action of several transcription factors acting in a time-specific manner. de Mattos et al. review current knowledge on the expression, function, and mechanism of action of various transcription factors that regulate fetal and adult endocrine Leydig cell development and function. The ability of Leydig cells to form androgen is influenced by other cells present in the interstitium, e.g., immune cells such as macrophages. Gu et al. review the literature showing that interstitial testicular macrophages are intimately associated with Leydig cells, controlling Leydig cell development and function, and that certain types of lymphocytes produce and metabolize steroids that might affect the steroidogenic output of the testis. Ruthig and Lamb review recent advances in understanding the interplay of Sertoli cell endocrine and paracrine signals that regulate germ cell state and thus, spermatogenesis. Recent studies of Sertoli cell ablation and transplantation that provide better clarity of the role of the Sertoli cell niche in germ cell development are discussed.

Sertoli cells are a major component of the spermatogonial stem cell niche and provide essential growth factors and chemokines to developing germ cells. Hoffman and McBeath review the activation of master regulators of the niche in Sertoli cells and their targets, and the molecular mechanisms underlying the regulation of growth and differentiation factors such as GDNF and retinoic acid by NOTCH signaling and other pathways.

Male fertility is reliant upon continuous production of sperm. Spermatogenesis involves the coordinated transitions of mitosis, meiosis, and spermiogenesis. Moritz and Hammoud review current understanding of chromatin dynamics during spermiogenesis, and the molecular basis of the histone-to-protamine exchange in idiopathic male infertility. The transition of Type A spermatogonia to differentiated spermatogonia requires the action of retinoic acid (RA). The synthesis of retinoic acid from retinal in the seminiferous epithelium is a result of the action of aldehyde dehydrogenases. Topping and Griswold report that of the three known retinal dehydrogenases involved in RA synthesis, two are required in Sertoli cells for normal spermatogenesis, and that the global deletion of the genes for these two enzymes blocks spermatogenesis, thus offering a potential target for contraception in the male.

Meyer-Ficca et al. address the question of whether age-related NAD+ decline is functionally linked to decreased male fertility. Using a transgenic mouse model, the authors report that decreasing testicular NAD+ levels in young adult mice, to levels that match or exceed the NAD+ decline observed in old mice, results in the disruption of spermatogenesis, and that providing vitamin B3 (niacin) to NAD+-depleted transgenic mice rescues spermatogenesis. The results suggest that NAD+ provided by vitamin B3 is important for complete spermatogenesis and male fertility.

Mundt et al. review publications on extracellular adenosine triphosphate (ATP) as a paracrine mediator of male fertility and sperm production, acting by targeting membrane-bound purinergic receptors and ion channels, and triggering changes in the cell’s membrane potential, calcium homeostasis, and cAMP levels. The summarized results demonstrate the importance of purinergic signaling in the control of male reproduction.

Kiyozumi and Ikawa describe biological processes regulated by proteases and protease inhibitors based on the use of gene-modified organisms. A focus is on the generation and activation of gametes during spermatogenesis. Discussed are proteolysis-related factors and biological processes regulated by proteolysis for successful reproduction, including cleavage of peptide bonds to activate and inactivate enzymes, transcription factors, and receptors.

## Effects of age and exogenous influences on sperm formation and function

Paternal age at conception has been steadily increasing globally. Chan and Robaire review results from mammalian animal models showing that increasing paternal age affects progeny outcome. Clinical studies reveal effects on offspring with respect to perinatal health, cancer risk, genetic diseases, and neurodevelopmental deficits. An overview of the potential mechanisms involved in altering germ cells in advanced age is presented. This is followed by an analysis of the current state of management of reproductive risks associated with advanced paternal age, and strategies for mitigating its impact.

Sakib et al. make the case that an *in vitro* system to study testicular maturation would serve as a platform for high-throughput drug and toxicity screening in a tissue-specific context. The authors report conditions that result in the successful generation and maintenance of rat testicular organoids in culture and the use of this system to study testicular cell maturation and the effects of exposure to toxicants.

Infection and inflammation can lead to infertility. The review by Hasan et al. describes evidence for the activation of inflammatory pathways as causative in various forms of male testicular disorders. The focus is on how imbalance of local testicular factors contributes to disturbances of spermatogenesis and steroidogenesis.

Wang et al. discuss the association of perinatal exposure to smoking and childhood asthma. They point out that although the molecular mechanisms underlying childhood asthma induced by perinatal exposure to smoking or nicotine remain elusive, an epigenetic mechanism might be involved. The new data presented in this paper show that perinatal exposure to nicotine leads to alterations in the profiles of sperm RNAs, including mRNAs and small RNAs, and that rosiglitazone, a PPARγ agonist, can reverse the negative effects on RNA.

The study by Starovlah et al. addresses the possible mechanism(s) by which acute psychological stress might affect male fertility in rat models. Included in the study are analyses of numbers of spermatozoa, markers of mitochondrial dynamics, and expression of signaling molecules.

Wang et al. discuss the X-linked miR-465 cluster. The study that the authors present focuses on the role of the miR-465 cluster in murine development. It is reported that ablation of the miR-465 miRNA cluster using CRISPR-Cas9 did not cause infertility, but rather a sex ratio biased toward males in the knockout mice. The data suggest that the miR-465 cluster is required for normal female placental development, and that ablation of the miR-465 cluster leads to a skewed sex ratio with more males (~60%) due to selective degeneration and resorption of the female conceptuses.

## Clinical applications: Testosterone, spermatogenesis and contraception

Hypogonadism and priapism have been shown to be common in men with sickle cell disease (SCD). Musicki and Burnett review the use of a mouse model for understanding the relationship of primary hypogonadism to SCD and to priapism. They also discuss the mechanisms involved in reduced cholesterol transport to and into the mitochondria in relationship to reduced testosterone, and how endogenous testosterone production might be restored specifically and safely in men with SCD, thereby reducing episodes of priapism.

Makela and Toppari review data showing that the retinoblastoma protein (RB) binds to E2F transcription factors in the testis, and that their interaction is a key mechanism involved in the establishment and maintenance of male fertility. In particular, evidence from gene knock-out studies is discussed that demonstrates that RB-E2F interaction in Sertoli cells is essential for fertility and is important for germline maintenance and lifelong sperm production.

Schlegel points out that much of what is understood about human spermatogenesis has come from the study of rodent models, but that this approach might not be ideal. This paper focuses on clinical observations of human spermatogenesis, focused mainly on genetic abnormalities in human sperm that are based on analyses conduct with patients presenting with symptoms of severe infertility.

Johnston and Lindsey discuss innovative approaches focused on expanding the contraceptive options available to men and women. They also consider new challenges to clinical development and regulatory approval, and how these challenges can be met so that new discoveries will move “from bench to bedside.”

In the last but not the least of these contributions, Page et al. emphasize the importance of effective contraceptive options for men and women and make the case for expanded male contraceptive methods. The authors discuss the use of exogenous progestins and androgen that suppress the hypothalamic-pituitary-gonadal axis as effective and reversible, and present new data on the use of novel steroids and varied routes of hormone delivery.

## Concluding remarks

The editors are sincerely grateful to all authors for their invaluable contributions highlighting current knowledge on spermatogenesis and its regulation by endocrine and paracrine factors. Moreover, we are grateful to the reviewers for their insightful and constructive reviews. Our hope is that these results are discussed, and the new techniques that now are available to investigators, will inspire further in-depth work in this important field of human biology. We also express our appreciation for the editorial assistance by the Frontiers staff and in particular that from Samuel Manning Journal Specialist, Frontiers in Endocrinology for his timely and helpful responses to our many questions during the entire project period.

## Author contributions

EG, PL, VP and BZ contacted potential authors, assigned manuscripts to reviewers, and ultimately made decisions as to the publication status of the manuscripts.

## Conflict of interest

The authors declare that the research was conducted in the absence of any commercial or financial relationships that could be construed as a potential conflict of interest.

## Publisher’s note

All claims expressed in this article are solely those of the authors and do not necessarily represent those of their affiliated organizations, or those of the publisher, the editors and the reviewers. Any product that may be evaluated in this article, or claim that may be made by its manufacturer, is not guaranteed or endorsed by the publisher.

